# Mucus Clearance Strategies in Mechanically Ventilated Patients

**DOI:** 10.3389/fphys.2022.834716

**Published:** 2022-03-23

**Authors:** Ryan L. Goetz, Kadambari Vijaykumar, George M. Solomon

**Affiliations:** ^1^Department of Medicine, Tinsley Harrison Internal Medicine Residency Program, University of Alabama at Birmingham, Birmingham, AL, United States; ^2^Division of Pulmonary, Allergy and Critical Care Medicine, University of Alabama at Birmingham, Birmingham, AL, United States; ^3^The Gregory Fleming James Cystic Fibrosis Research Center, University of Alabama at Birmingham, Birmingham, AL, United States

**Keywords:** mucus clearance, mucoactive therapies, cough augmenters, mechanical ventilation, extubation failure

## Abstract

The use of airway clearance strategies as supplementary treatment in respiratory disease has been best investigated in patients with cystic fibrosis (CF) and non-cystic fibrosis bronchiectasis (NCFBE), conditions which are traditionally characterized by excessive mucus stasis and mucociliary dysfunction. A variety of airway clearance therapies both pharmacological and non-pharmacological have been shown to ameliorate disease progression in this population and have hence been assimilated into routine respiratory care. This self-propagating cycle of mucus retention and airway damage leading to chronic inflammation and infections can also be applied to patients with respiratory failure requiring mechanical ventilation. Furthermore, excessive trachea-bronchial secretions have been associated with extubation failure presenting an opportunity for intervention. Evidence for the use of adjunctive mucoactive agents and other therapies to facilitate secretion clearance in these patients are not well defined, and this subgroup still remains largely underrepresented in clinical trials. In this review, we discuss the role of mucus clearance techniques with a proven benefit in patients with CF and NCFBE, and their potential role in patients requiring mechanical ventilation while highlighting the need for standardization and adoption of mucus clearance strategies in these patient populations.

## Introduction

Clearance of foreign particles and bacteria from the lungs is an important factor in host immunity, facilitated by the optimal functioning of the mucociliary system in conjunction with the cough reflex. The airway epithelium comprised of ciliated and secretory cells lines most of the conducting airways (Widdicombe and Wine, 2015). The secretory cells (including glands, goblet cells, and Clara cells) release various particles with antimicrobial and immunomodulatory properties including lysozyme, lactoferrin, proteases, and nitric oxide, in addition to mucins (Ganesan et al., 2013). Mucins (MUC5AC, MUC5B) are large adsorbent molecules binding water to form mucus and are believed to act as a scaffolding to protect and organize antimicrobial particles (Evans et al., 2010; Zanin et al., 2016). Disulfide bonds link these glycoproteins into large multi-multimers. Different subtypes of mucin have differing properties and predominate in different disease states (MUC5AC dominates in asthma; MUC5B dominates in chronic obstructive pulmonary disease (COPD) (Ehre et al., 2019). This resultant mucus layer coats the airways and adsorbs circulating particles and bacteria, which are propelled up by the cilia toward the oropharynx where it is swallowed (Wanner et al., 1996; Munkholm and Mortensen, 2014). When mucociliary clearance via ciliary mediated flow fails or is overwhelmed, cough is the next physiologic modality to propel particles, pathogens, and mucus out of the airways (Zahm et al., 1991; Knowles and Boucher, 2002).

There are two primary etiologies for a defective mucociliary clearance: dysfunctional cilia and dehydration of the airways. In primary ciliary dyskinesia (PCD) dysfunctional cilia fail to propel the mucus upward to the pharynx, while in CF dehydrated airways cause mucus to thicken with inability to be propelled up the airway to the pharynx (Chodhari et al., 2004; Fahy and Dickey, 2010). These genetic diseases represent severe examples of defective mucociliary clearance illustrating the principal of defective mucociliary clearance leading to recurrent infections (Chodhari et al., 2004; Fahy and Dickey, 2010).

Reduced mucociliary clearance has also been implicated in the pathogenesis of chronic airway infections, including: *Pseudomonas aeruginosa* (PsA), *Haemophilus influenzae*, and *Staphylococcus aureus* (Zhou-Suckow et al., 2017). PsA is the prototypical bacteria leading to chronic inflammation and defective mucociliary clearance via the release of proteases that induce epithelial damage and slow ciliary beat frequency (CBF) (Amitani et al., 1991). These chronic airway infections also lead to recruitment of neutrophils into the bronchial tree by a variety of different cytokines and chemotaxins, principally IL-8, and others including: IL-1β, tumor necrosis factor-α, and leukotriene B4 (Currie et al., 1987; Gaga et al., 1998; Mikami et al., 1998; Watt et al., 2004). Robust recruitment of inflammatory cells to the airways can lead to an inflammatory milieu in chronic lung diseases resulting in a cycle of overactive inflammation that actually impairs neutrophil phagocytic and killing function (particularly in PsA infection) (Berger et al., 1989; Hirche et al., 2008; King et al., 2009). This deficiency of airway clearance leading to chronic infections and inflammation has been referred to as the “vicious cycle” in CF and non-CF bronchiectasis (NCFBE), which is further perpetuated by an imbalance in repair and damage leading to destruction; a process that also likely occurs in mechanically ventilated patients (Cole, 1986).

Excessive tracheobronchial secretions along with an ineffective or weak cough have also been shown to be an important factor determining extubation failure in up to 89% of patients failing extubation or tracheostomy decannulation (Jaber et al., 2018; Maggiore et al., 2018). Evidence in support of airway clearance therapies for mechanically ventilated patients is sparse at best, underpinning the limitations for standardization of airway clearance protocols as a necessary part of routine care. In this review, we will explore mucus clearance techniques with proven benefit in patients with mucociliary disorders and explore their role in patients requiring mechanical ventilation.

## Airway Clearance Deficits in Critically Ill Patients

Mucociliary dysfunction occurs both in acutely and chronically mechanically ventilated patients for a multitude of reasons. We define direct effects as those resulting directly from the mechanical ventilation circuit (including epithelial damage from endotracheal tube and cuff, anesthetics, lack of humidification, direct cough impairment) vs. indirect effects resulting from comorbid critical illness (inflammation, immobility, atelectasis) (Sackner et al., 1975a; Konrad et al., 1994). Anesthetic medications, specifically dexmedetomidine and ketamine, have been shown in *in vitro* models to directly decrease mucociliary clearance (Feldman et al., 2021). High FiO2 is known to decrease tracheal mucus velocity, likely through impaired ciliary function (Sackner et al., 1975b). Endotracheal tube cuffs, through direct epithelial damage, have also been known to impair ciliary function and decrease tracheal mucus velocity (Sackner et al., 1975a).

**FIGURE 1:**
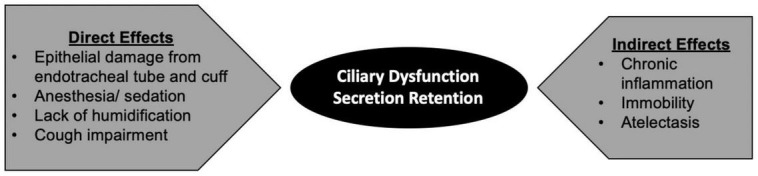
Direct and indirect mechanisms contributing to mucociliary impairment in mechanically ventilated patients.

Recurrent pulmonary infections may act as a predisposition among these patients to further exacerbate mucociliary dysfunction (Pittet et al., 2010; Locke et al., 2016). Cough is similarly impaired due to mechanical prevention of glottic closure by the endotracheal tube (Smina et al., 2003). Moreover, cough is further impaired by relative immobility and weakness, leading to increased atelectasis and secretion retention in this population (Ray et al., 1974; Schweickert and Hall, 2007; Figure 1).

## Treatment Modalities for Mucociliary Dysfunction in Critical Illness

**FIGURE 2:**
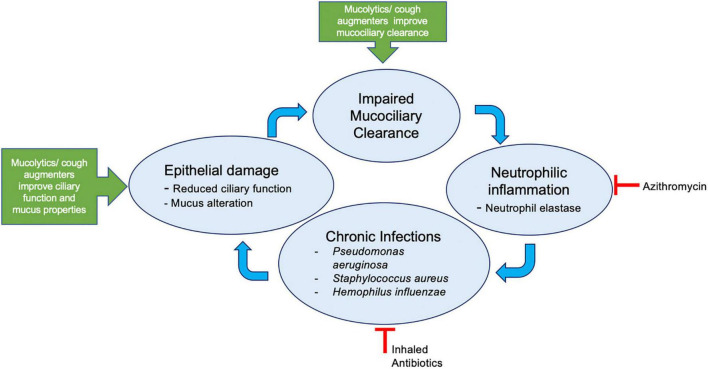
Deficiencies in airway clearance lead to self-propagating cycle of chronic infections and inflammation. Mucolytics, e.g., N-acetylcysteine, hypertonic saline, dornase alfa; cough augmenters, e.g., use of intrapulmonary percussive ventilation, high frequency chest wall oscillation, mechanical insufflation- exsufflation devices.

A theoretical framework of deficiencies in optimal airway clearance leading to the self-propagating cycle of chronic infections and inflammation is described in Figure 2. The available evidence for mucus clearance modalities in mechanically ventilated patients can be divided into mucoactive therapies including expectorants and mucolytics, cough augmentation strategies including high frequency chest wall oscillation (HFCWO) vest, oscillatory positive expiratory pressure (ex: acapella/flutter valve), mechanical insufflation-exsufflation (MI-E); and therapies directly targeting inflammation and infection.

## Therapies Addressing Underlying Mucus Alterations

Chronic inflammation and infection commonly lead to mucus derangements in patients undergoing mechanical ventilation (including patients with endotracheal tubes and/or tracheostomies) (Konrad et al., 1994). Therapies targeted to address these alterations are typically grouped into two main categories: mucolytic therapies, which are utilized to decrease the viscosity of mucus, with a more liquid secretion leading to ease of suctioning; and expectorant therapies, which are utilized to hydrate the mucus and increase cough to assist clearance. Efficacy data from CF and NCFBE has led to the testing of n-acetylcysteine and dornase alfa as mucolytic therapy and hypertonic saline as expectorant therapy in mechanically ventilated patients (Shah et al., 2005; Rogers, 2007).

Nebulized n-acetylcysteine (NAC) functions as a mucolytic by severing disulfide bonds that link mucin oligomers (Rogers, 2007). In vitro studies have demonstrated that NAC rapidly decreases mucus viscosity, however, it may increase the risk of bronchospasm due its acidic pH (pH = 2.2) (Sheffner et al., 1964). This risk may be mitigated by pre-treatment with a bronchodilator or by utilizing a reduced concentration of NAC (10% as opposed to 20% concentration) (Henke and Ratjen, 2007). Use of oral N-acetylcysteine has been investigated in adults with non-CF bronchiectasis with a reported reduction in risk of exacerbations. 24.7% of the patients in N-acetylcysteine group compared to 11.3% of the control group remained exacerbation-free throughout the 12-month treatment period (*P* = 0.026). Additionally, the volume of 24-h sputum in the N-acetylcysteine group was significantly reduced (*P* = 0.002) (Qi et al., 2019). Other newer agents can also address these disulfide bonds, such as P3001. P3001 has a lower pKa of the active moiety and more rapid kinetics of disulfide bond reduction (Ehre et al., 2019). Data supporting use of NAC as a mucolytic in the mechanically ventilated population is limited. Use of nebulized NAC (2 mL of 20% NAC diluted within 8 mL of normal saline administered three times daily for 1 day) in a randomized controlled trial of 40 mechanically ventilated patients found a lower mean secretion density and increased oxygen saturation but failed to demonstrate superiority in comparison to normal saline nebulization (Masoompour et al., 2015).

Dornase alfa is a highly purified solution of recombinant human deoxyribonuclease-I (rhDNase), an enzyme which selectively cleaves DNA polymers (Rubin, 2014). Dornase alfa decreases neutrophilic airway inflammation resulting in reduced frequency of respiratory exacerbations and to improved pulmonary function in CF (McCoy et al., 1996; Quan et al., 2001; Paul et al., 2004; Ratjen et al., 2005). Few studies have explored the effectiveness of dornase alfa in mucus clearance in mechanically ventilated patients. In one pediatric study, 100 infants undergoing mechanically ventilation following cardiac surgery were randomized to dornase alfa and placebo arms. Although no difference was found in the primary outcome of reintubation, the trial demonstrated shorter time on mechanical ventilation, decreased atelectasis, and decreased ICU stay in the dornase alfa arm. The regimen utilized in this study was 0.1-0.2 mg/kg twice daily from time of surgery until extubation (Riethmueller et al., 2006). Youness et al. (2012) conducted a placebo controlled double blind trial of 33 adult mechanically ventilated patients with new onset lobar or multi-lobar collapse. Patients were randomized to 7% hypertonic saline (HTS), dornase alfa, or normal saline (NS). Therapies were administered twice daily for seven days or until complete resolution of atelectasis. There was no statistically significant difference between groups, but a trend toward improvement in atelectasis scores was appreciated in the dornase alfa group (reduction by 2.18 points in imaging score versus 1.09 and 1.00 in HTS and NS groups, respectively). While the authors did not posit a physiological hypothesis for this trend, there was concern for the lack of a standardized atelectasis scoring system which contributed to missing (14%) of the patient data, thus affecting the statistical analysis. However, this data suggests that complex mucus treated with potent mucolytics should be better explored for mucus plugging resulting in atelectasis and prolonged ventilatory failure. A randomized placebo-controlled, double-blind pilot study of 30 patients examined dornase alfa 2.5 mg twice daily compared to placebo in patients with new onset atelectasis in acutely mechanically ventilated patients. Patients were treated until extubation, death, or transfer up to 30 days. There was no difference in chest X-ray scores (primary outcome), however the intervention group did show improved oxygenation and more extubation on treatment day 1 (Zitter et al., 2013).

Hypertonic saline has been used as both a mucolytic and an expectorant, and has shown to decrease bacterial biofilm formation (Elkins and Bye, 2011; Rubin, 2014). In patients admitted with CF exacerbation HTS has also shown to decrease time to clinical improvement of lung function and significantly reduce symptom burden at discharge (Dentice et al., 2016). The use of HTS in patients undergoing mechanical ventilation has not been extensively investigated; however, it is commonly utilized in clinical practice. A study randomized 18 pediatric patients undergoing mechanical ventilation to nebulized 3% HTS versus 0.9% saline four times daily until seven days or until extubation in a prophylactic manner for secretions. This study found a greater duration of mechanical ventilation amongst patients receiving HTS (Shein et al., 2016). Patients randomized to HTS group had markers of greater illness severity at baseline when compared to NS. It was also notable that this therapy was administered prophylactically early in the disease course. In this population, we believe that there would be little in the way of benefit for HTS and that the differences in duration of mechanical ventilation likely represent baseline differences in the populations rather than treatment effect.

Few studies have explored a combination therapy of dornase alfa and HTS. The study from 2012 discussed above randomized patients to 7% HTS, dornase alfa, or normal saline. It found no statistically significant difference between HTS and normal saline in chest x-ray scores or oxygenation (Youness et al., 2012). A retrospective case control cohort study evaluated the use of 7% HTS and dornase alfa in mechanically ventilated neonates with atelectasis who were unresponsive to conventional airway clearance methods. Both medications were given twice daily. All treatment arms experienced greater improvement in atelectasis compared to control (27% in control group, 70% with HTS, 81% with dornase alfa, and 95% with combination therapy); however there was not a statistically significant difference between individual arms (Altunhan et al., 2012).

Data from meta- analysis of 4 mucoactive agents (including NAC, hypertonic saline, heparin, and ambroxol) across 13 randomized control trials have shown no effect on the duration of mechanical ventilation or hospital stay, but the analysis demonstrated a small effect on reducing ICU length of stay in the mucoactive agent groups (10 trials, 95% CI −5.49 to -0.96, I^2^= 89%) was appreciated (Anand et al., 2020).

Taken together, this data suggests that there is reason to utilize mucolytic and expectorant therapies in patients requiring mechanical ventilation, especially in those with excessive secretions and approaching extubation. However, the available evidence in critically ill patients is based on anecdotal data and expert recommendations, rather than validated studies, which limits drawing a definitive conclusion for the standardization of treatment regimens in critically ill patients. These trial data are summarized in Supplementary Table 1.

## Cough Augmentation Strategies

In addition to the aforementioned deficits in airway clearance, cough and bulk mucus clearance are also altered in mechanically ventilated patients. Besides the mechanically prevented glottic closure, extensive and routine use of analgesics/sedative medications also suppress cough (Smina et al., 2003; Rosière et al., 2009). Impaired cough and excessive secretions notably have a prognostic significance for patients approaching liberation from the ventilator (Thille et al., 2015). Decreased and ineffective cough strength have also been associated with a higher risk of extubation failure (Thille et al., 2015; Gobert et al., 2017). A prospective, observational cohort study of 91 adult patients in medical-cardiac ICUs recovering from respiratory failure who passed a pressure support ventilation trial found that 89% of re-intubation cases have an excess of secretions vs. 39% of the successful extubations (Khamiees et al., 2001).

Cough assist devices, such as mechanical insufflation-exsufflation (MI-E), have also been explored in mechanically ventilated patients. Mechanical insufflation-exsufflation works on the principle that following deep inspiration with positive pressure, air in the lungs is expelled out immediately and rapidly by a forced negative pressure exsufflation. This resultant high pressure differential between the mouth and the alveoli creates a gradient to assist in the clearance of secretions (Homnick, 2007).

One single-center RCT of 180 adult ICU patients examined the use of MI-E in early mechanically ventilated patients which demonstrated improved secretion clearance (measured by weight of aspirated secretions) and lung compliance (Ferreira de Camillis et al., 2018).

High-frequency chest wall oscillation (HFCWO) involves oscillatory external chest wall compression causing expiratory flows that exceed inspiratory flow enhancing expectoration of mucus. In a pre-clinical canine model tracheal mucus transport was increased by 340% with HFCWO (King et al., 1983). This has resulted in the successful use of HFCWO in patients with CF and in patients with amyotrophic lateral sclerosis induced chronic respiratory failure (Warwick and Hansen, 1991; Lange et al., 2006). Use of HFCWO has mixed results in mechanically ventilated patients. In a recent study, 30 adult patients undergoing mechanical ventilation for acute respiratory failure were randomized to receive HFCWO or conventional chest physiotherapy (CCPT). In the HFCWO arm, patients had increased sputum weight, decreased atelectasis on imaging, improved oxygenation, and decreased tracheal culture positivity (Kuyrukluyildiz et al., 2016). Clinkscale et al. (2012) conducted a single center randomized trial that compared CCPT and HFCWO in a population including both mechanically ventilated and non-mechanically ventilated patients (CMVT). The study found no difference in length of hospital stay or duration of atelectasis but was underpowered for determining efficacy of either of these endpoints. Chuang et al. (2017) conducted a randomized trial of 73 patients mechanically ventilated for acute pneumonic respiratory failure comparing HFCWO and CCPT. They found decreased lower peak airway pressures following HFCWO, and higher oxygen saturations. This study was limited by methodologic concerns and multiple comparisons (Chuang et al., 2017). Additionally, a single center randomized study evaluating HFCWO vs. CCPT of 35 mechanically ventilated adults with COPD demonstrated a shorter total mechanical ventilation time in HFCWO group (10 days vs. 15) with no difference in invasive mechanical ventilation time, time in ICU or length of hospital stay (Liu et al., 2014).

Intrapulmonary percussive ventilation (IPV) is a newer modality in airway clearance. It generates “high flow mini-bursts of air along with a bronchodilator to the lungs at a rate of 300-400 times per minute”. This produces a higher pressure amplitude than unassisted modalities (e.g.; flutter valves and acapella devices) (Chatburn, 2007). IPV is commonly utilized in the outpatient setting in patients with CF and NCFBE (Natale et al., 1994). More recent studies have explored the use of IPV in mechanically ventilated patients. In a two-center RCT, 46 patients undergoing CMVT were randomized to IPV vs CCPT. After 15 days, the IPV group had significantly better oxygenation, higher maximal expiratory pressure, and a lower incidence of nosocomial pneumonia (Clini et al., 2006). A crossover study of 8 chronic tracheostomy patients (age 1-22 years) comparing IPV and HFCWO across various time periods showed significant reduction in the number of respiratory infections, need for systemic steroids, and hospitalizations with IPV (Bidiwala et al., 2017). Use of IPV was also evaluated in 10 obese mechanically ventilated patients with atelectasis in a retrospective case series which demonstrated improved oxygenation and lung compliance compared to case controls not receiving IPV (Tsuruta et al., 2006).

Taken together, this data suggests equipoise regarding the use of chest physiotherapy and other cough augmentation strategies, but there is insufficient data to conclude that any single modality is superior. These data are summarized in Supplementary Table 2.

## Modalities Directly Targeting Infection and Inflammation

Macrolide antibiotics are associated with a host of anti-inflammatory and immuno-modulatory effects achieved through multi-factorial mechanisms. One of the more commonly described pathways involves blockade of IL-8, leading to decreased airway neutrophil recruitment and neutrophil elastase elaboration (Verleden et al., 2006). Macrolides also reduce IL-1β concentrations, TNF- α, and IL-6 both in plasma and lung tissue. In mechanically ventilated patients, one RCT explored the role of azithromycin in PsA colonized patients undergoing mechanical ventilation and found a non-significant decrease in the incidence of VAP. Post hoc analysis showed greater decrease in VAP in patients with PsA strains with quorum sensing-dependent virulence factors (van Delden et al., 2012). A larger study randomized 200 patients with VAP to clarithromycin for 3 days vs. placebo (in addition to standard anti-microbial therapy determined by treating clinician). They demonstrated improved time to clinical resolution, reduced duration of mechanical ventilation, and delayed onset of multi-organ dysfunction (Giamarellos-Bourboulis et al., 2008). An analysis of cytokine levels during this study demonstrated an improved balance between pro-inflammatory versus anti-inflammatory mediators in patients treated with clarithromycin (Spyridaki et al., 2012). Thus, macrolide antibiotics may help directly address the excess inflammation which drives morbidity in this population.

Inhaled antibiotics represent a safe mechanism to deliver high doses of targeted antibiotics to the lungs. Benefits in outpatients with targetable infections suggests the potential for a role in improving outcomes in mechanically ventilated patients. A meta-analysis of 5 RCTs examining the use of prophylactic aerosolized antibiotics (including gentamicin, ceftazidime, tobramycin, and polymyxin B) in mechanically ventilated patients found a reduced rate of nosocomial pneumonia in patients randomized to receive aerosolized antibiotics (Falagas et al., 2006). Interestingly, these trials did not include patients with asymptomatic colonization, who based on the CF/NCFBE data are most likely to show a benefit (Döring et al., 2012; Orriols et al., 2015). Future trials may explore the use of aerosolized antibiotics in mechanically ventilated patients colonized with PsA and other pathogens and further investigate the role of inhalational antibiotics for episodes of ventilator-associated pneumonias and pathogen eradication attempts.

## Endpoints in Trials

The data presented herein demonstrates a plethora of chosen outcomes for both acute and chronic mechanical ventilation. Endpoints summarized in Table 1. Disparate outcomes without expert and scientific consensus pose a threat to definitive development of therapies aimed at mucoactive therapies in acutely ventilated patients. A recently conducted international consensus study outlined a set of core outcome measures that should be recorded in all clinical trials of interventions intended to modify the duration of ventilation for invasively mechanically ventilated patients in the ICU and included opinions from critical care physicians, ICU survivors, clinical trial investigators among others. These core outcomes included rates of extubation, reintubation, duration of mechanical ventilation, length of stay, health-related quality of life, and mortality (Blackwood et al., 2019). In addition to these variables, it would be prudent to propose that endpoints for the study of mucus clearance therapies in mechanically ventilated patients be grouped into sputum characteristics, physiologic endpoints, and patient-centered endpoints. Sputum characteristics to consider in clinical trials include sputum weight and density. Physiologic endpoints would include oxygenation (PaO_2_, pulse oximetry, and PaO_2_:FiO_2_), ventilation (PaCO_2_), ventilator physiology (peak and plateau airway pressures). Patient centered outcomes would include duration of mechanical ventilation, hospital length of stay, incidence of reintubation, occurrence of nosocomial pneumonia, and mortality. Determining appropriate clinical outcomes represents a challenge in this population given the significant heterogeneity of the patient population, poorly defined outcomes (including VAP), and difficulties identifying the best surrogate endpoints. However, since sputum characteristics, duration of mechanical ventilation, and reduction of VAP episodes seem most rigorously studied, we propose that these be a focus in developing therapies in the future from registry/observational studies to determine their role in surrogacy. Until further research is conducted, however, definitive conclusions from existing comparator studies will remain hampered in this patient population.

**TABLE 1:** Summary of endpoints in various listed trials and their comparative outcomes.

Trial, first author	Sputum Characteristics	Respiratory physiology	Ventilator physiology	Duration of Mechanical ventilation	Hospital/ICU length of stay	Nosocomial pneumonia	Mortality	Need for Reintubation
rhDNase Altunhan et al., 2012								
7% Hypertonic Saline+ rhDNase Altunhan et al., 2012								
7% Hypertonic Saline Youness et al., 2012								
rhDNase Youness et al., 2012								
3% Hypertonic Saline Shein et al., 2016				#				
N-acetyl cysteine Masoompour et al., 2015								
rhDNase Zitter et al., 2013								
rhDNase Pottecher et al., 2020		#						
rhDNase Riethmueller et al., 2006								
Cough Assist Ferreira de Camillis et al., 2018	#							
HFCWO Kuyrukluyildiz et al., 2016								
HFCWO Liu et al., 2014				#				
HFCWO g (Chuang et al., 2017			#					
HFCWO Clinkscale et al., 2012					#			
IPV Clini et al., 2006		#						
IPV Tsuruta et al., 2006								
IPV Toussaint et al., 2003								
IPV vs. HFCWO Bidiwala et al., 2017								

*Yellow: Intervention demonstrates benefit over comparator; Green: No difference in outcome between groups; Orange: Outcome worse in intervention Group; Gray: Measured but unclear (or pending) results, #: primary outcome.*

## Conclusion

Mucus clearance therapies form an essential part in the armamentarium available for the treatment of respiratory diseases associated with reduced or abnormal mucus clearance. Adoption of mucus clearance strategies in this critically ill population aims to break the self-propagating cycle of chronic inflammation/infections, and prevent extubation failures as a result of excessive tracheobronchial secretions. While both pharmacologic and non-pharmacologic modalities of airway clearance have been studied and are the standard of care in patients with CF and NCFBE, these interventions are still being explored as adjunctive therapeutic options in those requiring mechanical ventilation. Furthermore, trials conducted to date lack sufficient power, equipoise, and sufficient standardized endpoints to draw strong conclusions about therapy options. We postulate that it is beneficial to use mucoactive therapies in combination with cough augmentation strategies, however more studies with robust validation and standardized endpoints are needed to complement the existing expert recommendations.

## Author Contributions

RG, KV, and GS contributed equally to composition and editing of the manuscript. All authors contributed to the article and approved the submitted version.

## Conflict of Interest

The authors declare that the research was conducted in the absence of any commercial or financial relationships that could be construed as a potential conflict of interest.

## Publisher’s Note

All claims expressed in this article are solely those of the authors and do not necessarily represent those of their affiliated organizations, or those of the publisher, the editors and the reviewers. Any product that may be evaluated in this article, or claim that may be made by its manufacturer, is not guaranteed or endorsed by the publisher.
