# Supplementary material for: Mucus Clearance Strategies in Mechanically Ventilated Patients
Source: Front Physiol. 2022 Mar 23;13:834716. doi: 10.3389/fphys.2022.834716 (PMC8984116; doi:10.3389/fphys.2022.834716)
Supplement: Supplementary file 1 [file Table_1.DOCX]

| Table 1. (Supplemental) Pharmacologic airway clearance therapies | | | | | |
| --- | --- | --- | --- | --- | --- |
|  | Population | Design | Intervention | Outcomes | Result |
| Altunhan 2012 | 87 newborns with persistent atelectasis | Retrospective | Group 1: None  Group 2: 7% HTS  Group 3: rhDNase  Group 4: 7% HTS, rhDNase | Improvement in atelectasis  PaCO_2_, RR, FiO2 requirement, Peak inspiratory pressure | These parameters were improved in Groups 2-4.  Greatest improvement with group 4 |
| Youness 2012 | 33 adults mechanically ventilated patients with new onset lobar or multi-lobar collapse | Prospective RCT | Group 1: NS  Group 2: 7% HTS  Group 3: rhDNase | CXR score (primary)  Airway pressures, oxygenation, time to extubation, extubation during 7-day period | Greater overall reduction in rhDNase group (no statistically significant on pairwise comparison)  No other changes |
| Shein 2016 | 18 pediatric patients intubated <12 h, with expected duration of MV >48h | Prospective RCT | Group 1: 3% HTS  Group 2: NS | Duration of MV (Primary)  CXR score, PIP, f, V_T_, PEEP, FiO_2_, dead space, E_T_CO_2_, SpO_2_ | Duration of mechanical ventilation prolonged with HTS (208.1 vs 129.5 h), after adjustment for baseline differences in PEEP  No difference in outcomes minimal changes in all secondary outcomes. |
| Masoompour 2015 | 40 MV patients  15-90 year old adults | Prospective RCT | Group 1: NAC  Group 2: NS | Secretion density (primary)  Oxygenation, peak/plateau airway pressures, rhonchi | Lower mean secretion density in NAC Group  Increased O_2_ saturation, no difference in peak/plateau airway pressures |
| Zitter 2013 | 30 MV adults with lobar collapse | Prospective RCT | Group 1: rhDNase  Group 2: Placebo | CXR score, compliance  Oxygenation, extubation | No difference in primary outcomes  Improvement of oxygenation, more patients extubated in first 24 hours in intervention group (did not persist) |
| Pottecher 2020 | Mechanically ventilated trauma associated ARDS | Multi-center RCT | Dornase alfa vs placebo | Primary outcome: Oxygenation  Secondary outcomes: improvement in lung function, mortality, LOS (hospital/ICU), compliance, VAP, 30-day mortality, DAMPs/NETs plasma concentration | Trial underway |
| Riethmueller 2006 | 105 infants requiring mechanical ventilation after surgery for congenital heart surgery | Single center RCT | Dornase alfa vs placebo | Reintubation, incidence of atelectasis, median ventilation time, median PICU LOS, mean cost | Decreased incidence of atelectasis, median ventilation time, median PICU LOS, mean cost. No difference in reintubation rate |
| PaCO_2_, arterial partial pressure CO_2_; RR, respiratory rate, FiO_2_, fraction inspired O_2_; NS, normal saline; PEEP, positive end-expiratory pressure; ARDS, Acute Respiratory Distress Syndrome; LOS, length of stay; MV, mechanical ventilation; PIP, positive inspiratory pressure; f, ventilatory rate; CXR, chest x-ray; V_t_, tidal volume; ; EtCO_2_, end-tidal CO_2_; HTS, hypertonic saline; NAC, n-acetyl cysteine; SpO_2_, peripheral O_2_ saturation; PEEP, positive end-expiratory pressure; DAMPs, Damage-associated molecular patterns; NETs, neutrophil extracellular traps; PICU, pediatric intensive care unit. | | | | | |

| Table 2. (Supplemental) Non- pharmacologic airway clearance therapies | | | | | |
| --- | --- | --- | --- | --- | --- |
|  | Population | Design | Intervention | Outcomes | Result |
| Ferreira de Camillis 2018 | 180 mechanically ventilated patients, stable for >24 hours | Single center RCT | Cough assist/MI-E device vs standard therapy alone | Mean weight of aspirated airway mucus, compliance, Airway resistance, WOB | Higher mean weight of aspirated airway mucus, increased compliance; similar airway resistance and WOB |
| Kuyrukluyildiz 2016 | 30 mechanically ventilated patients | Single center RCT | Standard pulmonary rehab with CCPT vs HFCWO | APACHE-II scores, dry sputum weight, lung collapse index, PaO_2_, tracheal aspirate culture | No change in: APACHE score, Dry sputum weight, lung collapse index decreased,  PaO_2_ increased  Culture positivity at 72 hours decreased |
| Lui 2014 | 35 mechanically ventilated COPD patients | Single center RCT | HFCWO vs control | Duration of mechanical ventilation (total, invasive, non-invasive, ICU stay, hospital), pH, SpO2, peak airway pressure | Decreased total duration of mechanical ventilation  No difference in invasive and non-invasive, ICU stay, hospital stay.  No change in pH, SpO2, peak airway pressure |
| Chuang 2017 | 73 patients with mechanically ventilated with pneumonic respiratory | Single center RCT | HFCWO vs control | Mean airway pressure, respiratory rate, rapid shallow breathing index, SpO2 | Mean airway pressure, respiratory rate, rapid shallow breathing index increased  SpO2 decreased |
| Clinkscale 2012 | 280 patients requiring chest physiotherapy, included intubated and non-intubated | Single center RCT | HFCWO vs conventional CPT | Hospital stay, patient comfort, time to radiographic resolution of lobar atelectasis, hospital mortality, nosocomial pneumonia | Patient comfort greater with CCPT  Trend toward improved Lobar atelectasis and shorter duration of MV with CCPT |
| Clini 2006 | 46 tracheostomized patients in weaning center | Two center RCT | IPV vs CCPT | Arterial blood gas, P/F, maximal expiratory pressure, incidence of pneumonia | Improved oxygenation, decreased rate of nosocomial pneumonia |
| Tsuruta 2006 | 10 obese patients with respiratory failure | Single center cohort, retrospective | IPV | Oxygenation, compliance, HR, MAP, atelectasis on CT | Improved oxygenation (P/F 189 to 243 within 3 hours); improved dynamic compliance.  No change in HR or MAP |
| Toussaint 2003[78] | 8 patients with Duchenne Muscular Dystrophy s/p tracheostomy | Randomized cross-over study | IPV vs forced expiration and manual assisted cough | Weight of secretions, HR, RR, SpO2, EtCO2, airway resistance, peak expiratory flow | Increased mean weight of secretions, no change in other measures |
| Bidiwala 2017 | 8 tracheostomy dependent patients age 1-22 | Retrospective cross-over study | IPV vs HFCWO | Total # of respiratory illnesses, LRTI requiring antibiotics, use of bronchodilator treatments, utilization of systemic steroids, number of hospitalizations | All outcomes Improved during IPV period |
| MI-E, mechanical insufflation-exsufflation; WOB, work of breathing; HFCWO, high frequency chest wall Oscillation ventilation; CCPT, chest physiotherapy; APACHE, Acute Physiology and Chronic Health Evaluation; PaO2, arterial partial pressure O_2_; SpO_2_, peripheral O_2_ saturation; RR, respiratory rate; MAP, mean arterial pressure; HR, heart rate; EtCO_2_, end-tidal CO_2_; LRTI, lower respiratory tract infection; IPV, intrapulmonary percussive ventilation | | | | | |
